# Impact of Missing Value Imputation on Classification for DNA Microarray Gene Expression Data—A Model-Based Study

**DOI:** 10.1155/2009/504069

**Published:** 2010-01-04

**Authors:** Youting Sun, Ulisses Braga-Neto, EdwardR Dougherty

**Affiliations:** 1 Department of Electrical and Computer Engineering, Texas A&M University, College Station, TX 77843, USA; 2 Computational Biology Division, Translational Genomics Research Institution, Phoenix, AZ 85004, USA; 3 Department of Bioinformatics and Computational Biology, University of Texas M.D. Anderson Cancer Center, Houston, TX 77030, USA

## Abstract

Many missing-value (MV) imputation methods have been developed for microarray data, but only a few studies have investigated the relationship between MV imputation and classification accuracy. Furthermore, these studies are problematic in fundamental steps such as MV generation and classifier error estimation. In this work, we carry out a model-based study that addresses some of the issues in previous studies. Six popular imputation algorithms, two feature selection methods, and three classification rules are considered. The results suggest that it is beneficial to apply MV imputation when the noise level is high, variance is small, or gene-cluster correlation is strong, under small to moderate MV rates. In these cases, if data quality metrics are available, then it may be helpful to consider the data point with poor quality as missing and apply one of the most robust imputation algorithms to estimate the true signal based on the available high-quality data points. However, at large MV rates, we conclude that imputation methods are not recommended. Regarding the MV rate, our results indicate the presence of a peaking phenomenon: performance of imputation methods actually improves initially as the MV rate increases, but after an optimum point, performance quickly deteriorates with increasing MV rates.

## 1. Introduction

Microarray data frequently contain missing values (MVs) because imperfections in data preparation steps (e.g., poor hybridization, chip contamination by dust and scratches) create erroneous and low-quality values, which are usually discarded and referred to as missing. It is common for gene expression data to contain at least 5% MVs and, in many public accessible datasets, more than 60% of the genes have MVs [1]. Microarray gene expression data are usually organized in a matrix form with rows corresponding to the gene probes and columns representing the arrays. Trivial methods to deal with MVs in the microarray data matrix include replacing the MV by zero (given the data being in log domain) or by row average (RAVG). These methods do not make use of the underlying correlation structure of the data and thus often perform poorly in terms of estimation accuracy. Better imputation techniques have been developed to estimate the MVs by exploiting the observed data structure and expression pattern. These methods include K-nearest Neighbor imputation (KNNimpute) and singular value decomposition- (SVD-) based imputation [2], Bayesian principal components analysis (BPCA) [3], least square regression-based imputation [4], local least squares imputation (LLS) [5], and LinCmb imputation [6], in which the MV is calculated by a convex combination of the estimates given by several existing imputation methods, namely, RAVG, KNNimpute, SVD, and BPCA. In addition, a nonlinear PCA imputation based on neural networks was proposed for effectively dealing with nonlinearly structured microarray data [7]. Gene ontology-based imputation utilizes information on functional similarities to facilitate the selection of relevant genes for MV estimation [8]. Integrative MV estimation method (iMISS) aims at improving the MV estimation for datasets with limited numbers of samples by incorporating information from multiple microarray datasets [9].

In most of the studies about MV imputation, the performance of various imputation algorithms is compared in terms of the normalized root mean squared error (NRMSE) [2], which measures how close the imputed value is to the original value. However the problem is that the original value is unknown for the missing data, thus calculating NRMSE is infeasible in practice. To circumvent this problem, all the studies involving NRMSE calculation adopted the following scheme [2, 4–6, 9–11]: first, a subcomplete matrix is extracted from the original MV-contained gene expression matrix; then, entries of the complete matrix are randomly removed to generate the artificial MVs; Finally, MV imputation is applied. The NRMSE can now be calculated to measure the imputation accuracy, since the original values are now known. This method is problematic for two reasons. First, the selection of artificial missing entries is random and thus is independent of the data quality—whereas imputing data spots with low quality is the main scenario in real world. Secondly, in the calculation of the NRMSE, the imputed value is compared against the original, but the original is actually a noised version of the true signal value, and not the true value itself.

While much attention has been paid to the imputation accuracy measured by the NRMSE, a few studies have examined the effect of imputation on high-level analyses (such as biomarker identification, sample classification, and gene clustering), which demand that the dataset be complete. For example, the effect of imputation on the selection of differentially expressed genes is examined in [6, 11, 12] and the effect of KNN imputation on hierarchical clustering is considered in [1], where it is shown that even a small portion of MVs can considerably decrease the stability of gene clusters and stability can be enhanced by applying KNN imputation. The effects of various MV imputation methods on the gene clusters produced by the K-means clustering algorithm are examined in [13], the main findings being that advanced imputation methods such as KNNimpute, BPCA, and LLS yield similar clustering results, although the imputation accuracies are noticeably different in terms of NRMSE. To our knowledge, only two studies have investigated the relationship between MV imputation of microarray data and classification accuracy.

Wang et al. study the effects of MVs and their imputation on classification performance and report no significant difference in the classification accuracy results when KNNimpute, BPCA, or LLS are applied [14]. Five datasets are used: a lymphoma dataset with 20 samples, a breast cancer dataset with 59 samples, a gastric cancer dataset with 132 samples, a liver cancer dataset with 156 samples, and a prostate cancer dataset with 112 samples. The authors consider how differing amounts of MVs may affect classification accuracy for a given dataset, but rather than using the true MV rate, they use the MV rate threshold (MVthld) throughout their study, where, for a given MVthld (MVthld , where ), the genes with MV rate less than MVthld are retained to design the classifiers. As a result, the true MV rate (which is not reported) of the remaining genes does not equal MVthld and, in fact, can be much less than MVthld. Hence, the parameter MVthld may not be a good indicator. Moreover, the authors plot the classification accuracies against a number of values for MVthld, but as MVthld increases, the number of genes retained to design the classifier becomes larger and larger, so that the increase or decrease in the classification accuracy may be largely due to the additional included genes (especially if the genes are marker genes) and may only weakly depend on MVthld. This might explain the nonmonotonicity and the lack of general trends in most of the plots.

By studying two real cancer datasets (SRBCT dataset with 83 samples of 4 tumor types, GLIOMA dataset with 50 samples of 4 glioma types), Shi et al. report that the gaps between different imputation methods in terms of classification accuracy increase as the MV rate increases [15]. They test 5 imputation methods (RAVG, KNNimpute, SKNN, ILLS, BPCA ), 4 filter-type feature selection methods (-test, -test, cluster-based -test, and cluster-based F-test) and 2 classifiers (5NN and LSVM). They have two main findings: () when the MV rate is small (), all imputed datasets give similar classification accuracies that are close to that of the original complete dataset; however, the classification performances given by different datasets diverge as the MV rate increases, and () datasets imputed by advanced imputation methods (e.g., BPCA) can reach the same classification accuracy as the original dataset. A fundamental problem with their experimental design is that the MVs are randomly generated on the original complete dataset, which is extracted from the MV-contained gene expression matrix. Although this randomized MV generating scheme is widely used, it ignores the underlying data quality.

A critical problem within both aforementioned studies is that all training data and test data are imputed together before classifier design and cross-validation is adopted for the classification process. The test data influences the training data in the imputation stage and the influence is passed to the classifier design stage. Therefore, the test data are involved in the classification design process, which violates the principle of cross-validation.

In this paper, we carry out a model-based analysis to investigate how different properties of a dataset influence imputation and classification, and how imputation affects classification performance. We compare six popular imputation algorithms, namely, RAVG, KNNimpute, LLS.L2, LLS.PC, LS, and BPCA, by measuring how well the imputed dataset can preserve the discriminant power residing in the original dataset. An empirical analysis using real data from cancer microarray studies is also carried out. In addition, the NRMSE-based comparison is included in the study, with a modification in the case of the synthetic data to give an accurate measure. Recommendations for the application of various imputations under different situations are given in Section 3.

## 2. Methods

### 2.1. Model for Synthetic Data

Many studies have shown the log-normal property of microarray data, that is, the distribution of log-transformed gene expression data approximates a normal distribution [16, 17]. In addition, biological effects which are generally assumed to be multiplicative in the linear scale become additive in the log scale, which simplifies data analysis. Thus, the ANOVA model [18, 19] is widely used, in which the log-transformed gene expression data are represented by a true signal plus multiple sources of additive noise.

There are other models proposed for gene expression data, including a multiplicative model for gene intensities [20], a hierarchical model for normalized log ratios [21], and a binary model [22]. The first two of these models do not take gene-gene correlation into account. In addition, the second model does not model the error sources. The binary model is too simplistic and not sufficient for the MV study in this paper.

Based on the log-normal property and inspired by ANOVA, we propose a model for the normalized log-ratio gene expression data which is centered at zero, assuming that any systematic dependencies of the log-ratio values on intensities have been removed by methods such as Lowess [23, 24]. Here, we consider two experimental conditions for the microarray samples (e.g., mutant versus wild-type, diseased versus normal). The model can be easily extended to deal with multiple conditions as well.

Let  be the gene expression matrix with  genes (rows) and  array samples (columns).  denotes the log-ratio of expression intensity of gene  in sample  to the intensity of the same gene in the baseline sample.  consists of the true signal  plus additive noise : (1)

The true signal is given by(2)

where  represents the log-transformed fold change and  is a term introduced to create correlation among the genes.

The log-transformed fold-change  is given by(3)

under the constraint that  is constant across all the samples in the same class. The parameters  and  are picked from a univariate Gaussian distribution, , where the mean log-transformed fold change  is set to 0.58, corresponding to a 1.5-fold change in the original linear scale, as this is a level of fold change that can be reliably detected [20]. The standard deviation of log-transformed fold change  is set to 0.1.

The distribution of  is multivariate Gaussian with mean 0 and covariance matrix . A block-based structure [25] is used for the covariance matrix to reflect the interactions among gene clusters. Genes within the same block (e.g., genes belong to the same pathway) are correlated with correlation coefficient  and genes within different blocks are uncorrelated as given by the following equation: (4)

where(5)

In the above equations, the gene block standard deviation , correlation , and size  are tunable parameters, the values of which are specified in Section 3.

The additive noise  in (1) is assumed to be zero-mean Gaussian, . The standard deviation  varies from gene to gene and is drawn from an exponential distribution with mean  to account for the nonhomogeneous missing value distribution generally observed in real data [26]. The noise level  is a tunable parameter, the value of which is specified in Section 3.

Following the model above, we generate synthetic gene expression datasets for the true signal, , and the observed expression values, . In addition, the dataset with MVs  is generated by identifying and discarding the low-quality entries of , according to(6)

The threshold  is adjusted to give varying rates of missing values in the simulated dataset, as discussed in Section 3.

### 2.2. Imputation Methods

Following the notation of [27], a gene with MVs to be estimated is called a target gene, with expression values across array samples denoted by the vector . The observable part and the missing part of  are denoted by  and , respectively. The set of genes used to estimate  forms the candidate gene set  for .  is partitioned into  and  according to the observable and the missing indexes of . In row average imputation (RAVG), the MVs of the target gene  are simply replaced by the average of observed values, that is, .

We will discuss three more complex methods, namely, KNNimpute, LLS, and LS imputation, which follow the same two basic steps.

(1) For each target gene ,  genes with expression profiles most similar to the target gene are selected to form the candidate gene set .

(2) The missing part of the target gene  is estimated by a weighted combination of the corresponding  candidate genes . The weights are calculated in different manners for different imputation methods.

We will additionally describe briefly the BPCA imputation method.

#### 2.2.1. K-Nearest Neighbor Imputation (KNNimpute)

In the first step, the  norm is employed as the similarity measure for selecting the  neighbor genes (candidate genes). In the second step, the missing part of the target gene () is estimated as a weighted average (convex combination) of the corresponding parts of the candidate genes () which are not allowed to contain MVs at the same positions as the target gene:(7)

The weight for each candidate gene is proportional to the reciprocal of the  distance between the observable part of the target () and the corresponding part of the candidate ():(8)

where(9)

The performance of KNNimpute is closely associated with the number of neighbors  used. A value of  within the range of 10–20 was empirically recommended, while the performance (in terms of NRMSE) degraded when  was either too small or too large [2]. We use the default value of  in Section 3.

#### 2.2.2. Local Least Squares Imputation (LLS)

In the first step, either the  norm or the absolute value of the Pearson correlation coefficient is employed as the similarity measure for selecting the  candidate genes [5], resulting in two different imputation methods LLS.L2 and LLS.PC, respectively, with the former reported to perform slightly better than the latter. Owing to the similarity of performance, for clarity of presentation we only show LLS.L2 in the results section (the full results including LLS.PC are given on the companion website http://gsp.tamu.edu/Publications/supplementary/sun09a).

In the second step, the missing part of the target gene is estimated as a linear combination (which need not be a convex combination) of the corresponding parts of its candidate genes (whose MVs are initialized by RAVG):(10)

where the vector of weights  solves the least squares problem: (11)

As is well known, the solution is given by(12)

where  denotes the pseudo inverse of matrix .

#### 2.2.3. Least Squares Imputation (LS)

In the first step, similar to LLS.PC, the  most correlated genes are selected based on their absolute correlation to the target gene [4].

In the second step, the least squares estimate of the target given each of the  candidate gene is obtained:(13)

where the regression coefficient  is given by(14)

where  denotes the sample covariance between the target  and the candidate  and  is the sample variance of the candidate .

The missing part of the target gene is then approximated by a convex combination of the  single regression estimates:(15)

The weight of each estimate is a function of the correlation between the target and the candidate gene:(16)

The normalized weights are then given by .

#### 2.2.4. Bayesian Principal Component Analysis (BPCA)

BPCA is built upon a probabilistic PCA model and employs a variational Bayes algorithm to iteratively estimate the posterior distribution for both the model parameters and the MVs until convergence. The algorithm consists of three primary processes, which are (1) principle component regression, (2) Bayesian estimation, and (3) an expectation-maximization-like repetitive algorithm [3]. The principal components of the gene expression covariance matrix are included in the model parameters, and redundant principal components can be automatically suppressed by using an automatic relevance determination (ARD) prior in the Bayes estimation. Therefore, there is no need to choose the number of principal components one wants to use, and the algorithm is parameter free. We refer the reader to [3] for more details.

### 2.3. Experimental Design

#### 2.3.1. Synthetic Data

Based on the previously described data model, we generate various synthetic microarray datasets by changing the values of the model parameters, corresponding to various noise levels, gene correlations, MV rates, and so on (more details are given in Section 3). The MVs are determined by (6), with the threshold  adjusted to give a desired MV rate. For each of the models, the simulation is repeated  times. In each repetition, according to (1) and (2), the true signal dataset, , and the measured-expression dataset, , are first generated. The dataset  with missing values is then generated based on the data quality of  and a given MV rate. Next, six imputation algorithms, namely, RAVG, KNNimpute, LLS.L2, LLS.PC, LS, and BPCA are applied separately to calculate the MVs, yielding six imputed datasets, , for . Each of these training datasets contains  genes and  array samples and is used to train a number of classifiers separately. For each , a measured-expression test dataset  and a missing value dataset  are generated independently of, but in an identical fashion to, the datasets  and , respectively. Each of these test sets contains  genes and  array samples,  being large in order to achieve a very precise estimate of the actual classification error.

A critical issue concerns the manner in which the test data are employed. As noted in the introduction, imputation cannot be applied to the training and test data as a whole. Not only does this make the designed classifier dependent on the test data, it also does not reflect the manner in which the classifier will be employed. Testing involves a single new example, independent of the training data, being labeled by the designed classifier. Thus, error estimation proceeds in the following manner after imputation has been applied to the training data and a classifier designed from the original and imputed values: () an example  is selected and adjoined to the measured-expression training set ; () missing values are generated to form the set  [note that ]; () imputation is applied to , the purpose being to utilize the training data in the imputation for  to obtain the complete vector  (the superscript  means one imputation method); (4) the designed classifier is applied to  and the error ( or ) recorded; (5) the procedure is repeated for all test points; and (6) the estimated error is the total number of errors divided by . Notice that the training data are used in the imputation for the newly observed example, which is part of the classifier. The classifier consists of imputation for the newly observed example following by application of the classifier decision procedure, which has been designed on the training data, independently of the testing example. Overall, the classifier operates on the test example in a manner determined independently of the test example. If the imputation for the test data were independent of the training data, then one would not have to consider imputation as part of the classification rule; however, when the imputation for the test data is dependent on the training data, it must be considered part of the classification rule.

The classifier training process includes feature selection, and classifier design based on a given classification rule. Three popular classification rules are used in this paper: Linear Discriminant Analysis (LDA), 3-Nearest Neighbor (3NN) and Linear Support Vector Machine (LSVM)[28]. Two feature selection methods, -test and sequential forward floating search (SFFS)[29], are considered in our simulation study. The former is a typical *filter* method (i.e., it is classifier-independent) while the latter is a standard procedure used in the *wrapper* method (i.e., it is associated with classifier design and is thus classifier-specific). SFFS is a development of the sequential forward selection(SFS) method. Starting with an empty set , SFS iteratively adds new features to , so that the new set  is the best (gives the lowest classification error) among all . The problem with SFS is that a feature added to A early may not work well in combination with others but it cannot be removed from A. SFFS can mitigate the problem by **"**looking-back" for the features already in set . A feature is removed from  if  is the best among all , unless , called the "least significant feature", is the most recently added feature. This exclusion continues, one feature at a time, as long as the feature set resulting from removal of the least significant feature is better than the feature set of the same size found earlier in the SFFS procedure [30]. For the wrapper method SFFS, we use bolstered error estimation [31]. In addition, considering the intense computation load requested by SFFS in the high-dimension problems such as microarray classification, a two-stage feature selection algorithm is adopted, in which the -test is applied in the first stage to remove most of the noninformative features and then SFFS is used in the second stage [25]. This two-stage scheme takes advantage of both the filter method and the wrapper method and may even find a better feature subset than directly applying the wrapper method to the full feature set [32]. In summary, for each of the data models, 8 pairs of training and testing datasets are generated and are evaluated by a combination of 2 feature selection algorithms and 3 classification rules, resulting in a very large number of experiments.

**Figure 1:**
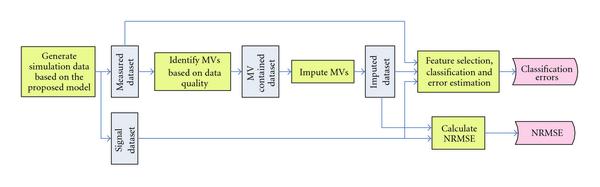
**Simulation flow chart**.

Each experiment is repeated 150 times, and the average classification error is recorded. The averaged classification error plots for different datasets, feature selection methods and classification rules are shown in Section 3. Besides the classification errors, the NRMSE between the signal dataset and each of the 6 imputed datasets is also recorded. The simulation flow chart is shown in Figure 1.

As previously mentioned, there can be drawbacks associated with the NRMSE calculation; however, in our simulation study, the MVs are marked according to the data quality and the NRMSE is calculated based on the true signal dataset which can serve as the ground truth: (17)

In this way, the aforementioned drawbacks about using NRMSE are addressed.

#### 2.3.2. Patient Data

In addition to the synthetic data described in the previous section, we used the two following publicly available datasets from published studies.

(i) Breast Cancer Dataset (BREAST)

Tumor samples from 295 patients with primary breast carcinomas were studied by using inkjet-synthesized oligonucleotide microarrays which contained 24,479 oligonucleotides probes along with 1281 control probes [33]. The samples are labeled into two groups [34]: 180 samples for poor-prognosis signature group, and 115 samples for good-prognosis signature. In addition to the log-ratio gene expression data, the log error data is also available which can be used to assess the data quality.

(ii) Prostate Cancer Dataset (PROST)

Samples of 71 prostate tumors and 41 normal prostate tissues were studied, using cDNA microarray containing 26,260 different genes [35]. In addition to the log-ratio gene expression data, additional information such as background (foreground) intensities and SD of foreground and background pixel intensities are also available and thus can be used to calculate the log error according to the Rosetta error model [36]—the log error  for the th probe in the th microarray sample is given by the following equation: (18)

where (19)

In the above equations,  specifies the red or green channel in the two-dye experiment,  and  denote the SD of foreground and background pixels, respectively, of the th probe in the th microarray sample,  and  are the numbers of pixels used in the mean foreground and background calculation, respectively, and  and  are the mean foreground and background intensities, respectively.

For the patient data study, the schemes used for imputation, feature selection and classification are similar to those applied in the synthetic data simulation, except that we use hold-out-based error estimation, that is, in each repetition,  samples are randomly chosen from all the samples as the training data and the remaining  samples are used to test the trained classifiers, with  being much larger than  in order to make error estimation precise. We preprocess the data by removing genes which have an unknown or invalid data value in at least one sample (flagged manually and by the processing software). After this preprocessing step, the dataset is complete, with all data values being known. We further preprocess the data by filtering out genes whose expressions do not vary much across all the array samples [13, 35]; indeed, the genes with small expression variance do not have much discrimination power for classification and thus are unlikely to be selected by any feature selection algorithm [15]. The resulting feature sizes are 400 and 500 genes for the prostate and the breast dataset, respectively. It is at this point where we begin our experimental process by generating the MVs.

Unlike the synthetic study, the true signal dataset is unknown in the patient data study since the data values are always contaminated by measurement errors. Therefore, in the absence of the true signal dataset, the NRMSE is calculated between the measured dataset and each of the imputed datasets (which is the usual procedure adopted in the literature). Thus the NRMSE result is less reliable in the patient data study, which highlights further the need for evaluating imputation on the basis of other factors, such as classification performance.

## 3. Results

### 3.1. Results for the Synthetic Data

**Figure 2:**
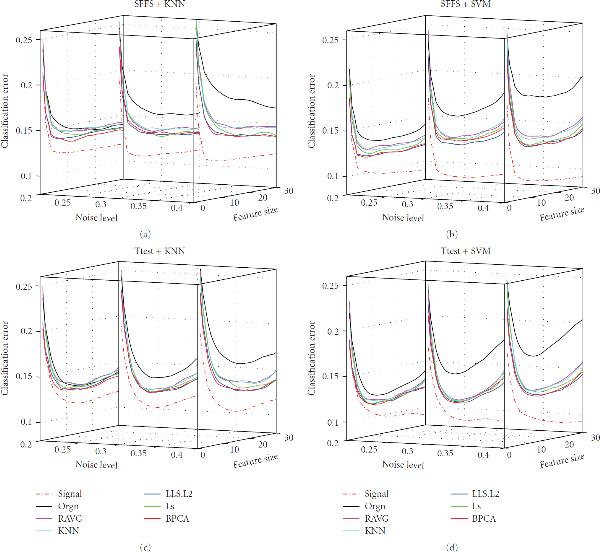
**Effect of noise level**. The classification error of the signal dataset (signal), the measured dataset (orgn), and the five imputed datasets. The underlying distribution parameters are SD , gene correlation , MV rate . Each panel in the figure corresponds to one combination of the feature selection methods and the classification rules, which is given by the title. The -axis labels the number of selected genes, the -axis is the noise level, and the -axis is the classification error.

**Figure 3:**
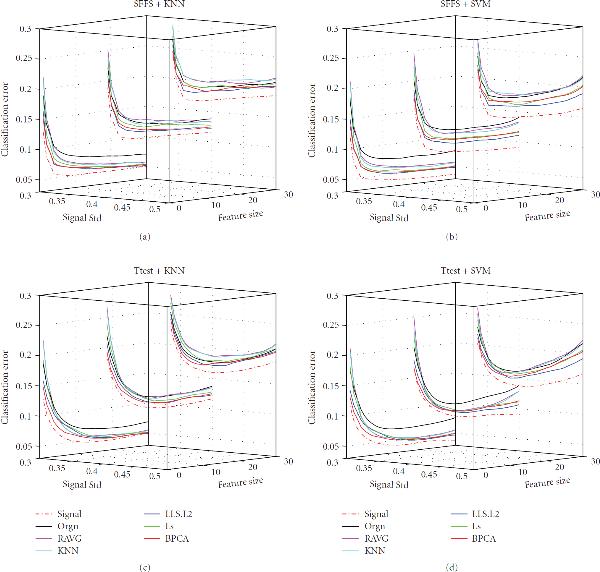
**Effect of variance**. The classification error of the signal dataset (signal), the measured dataset (orgn), and the five imputed datasets. The underlying distribution parameters are noise level , gene correlation , MV rate . Each panel in the figure corresponds to one combination of the feature selection methods and the classification rules, which is given by the title. The -axis labels the number of selected genes, the -axis is the signal SD, and the -axis is the classification error.

**Figure 4:**
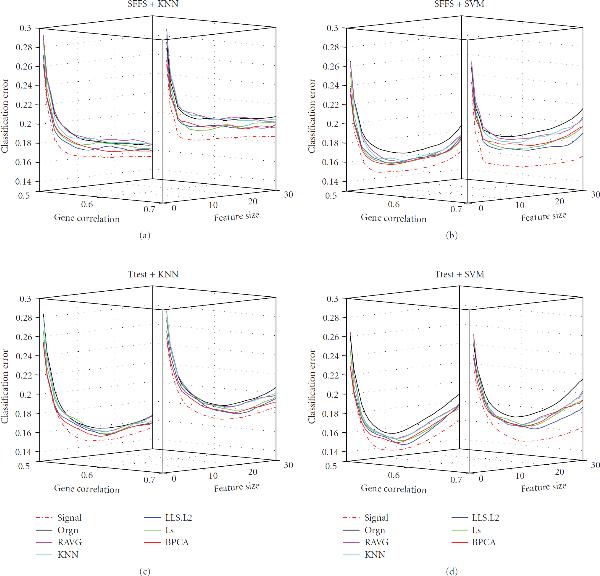
**Effect of correlation**. The classification error of the signal dataset (signal), the measured dataset (orgn), and the five imputed datasets. The underlying distribution parameters are SD , noise level , MV rate . Each panel in the figure corresponds to one combination of the feature selection methods and the classification rules, which is given by the title. The -axis labels the number of selected genes, the -axis is the gene correlation strength, and the -axis is the classification error.

**Figure 5:**
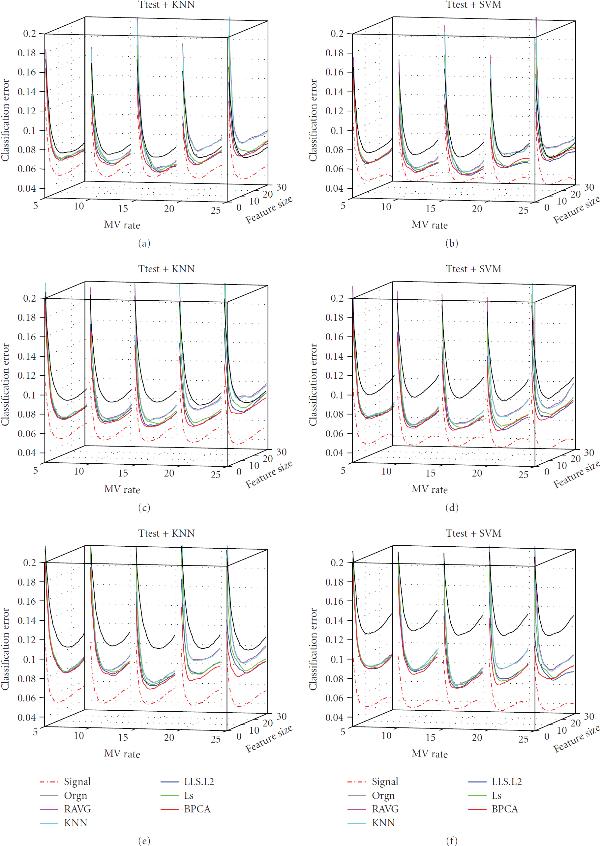
**Effect of MV Rate**. The classification error of the signal dataset (signal), the measured dataset (orgn), and the five imputed datasets. The underlying distribution parameters are SD , gene correlation , and noise level  for subfigures (a), (b), (c), (d), (e), and (f), respectively. The -axis labels the number of selected genes, the -axis is the MV rate, and the -axis is the classification error.

**Figure 6:**
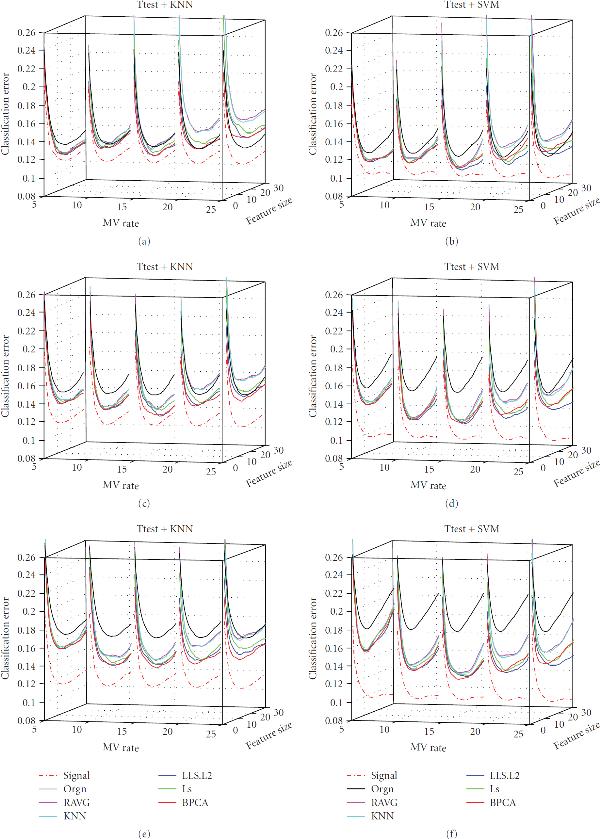
**Effect of MV Rate**. The classification error of the signal dataset (signal), the measured dataset (orgn), and the five imputed datasets. The underlying distribution parameters are SD , gene correlation , and noise level  for subfigures (a), (b), (c), (d), (e), and (f), respectively. The -axis labels the number of selected genes, the -axis is the MV rate, and the -axis is the classification error.

**Figure 7:**
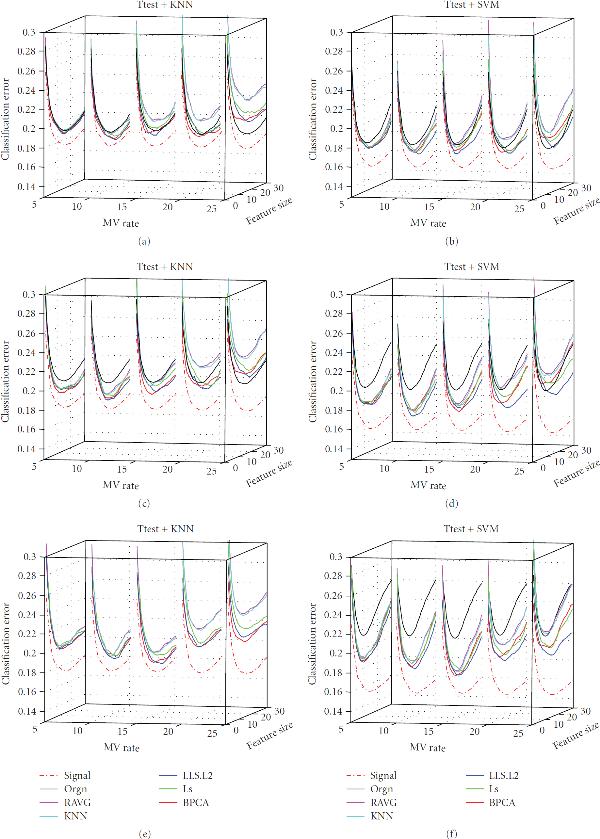
**Effect of MV Rate**. The classification error of the signal dataset (signal), the measured dataset (orgn), and the five imputed datasets. The underlying distribution parameters are SD , gene correlation , and noise level  for subfigures (a), (b), (c), (d), (e), and (f), respectively. The -axis labels the number of selected genes, the -axis is the MV rate, and the -axis is the classification error.

We have considered the model described in the previous section, for different combinations of parameters, which are displayed in Table 1. In addition, since the signal dataset is noise-free, the classification performance given by the signal dataset can serve as a benchmark. In the other direction, the benefit of an imputation algorithm is determined by how well imputation improves the classification accuracy of the measured dataset. The classification errors of the true signal dataset, measured dataset, and imputed datasets under different data distributions are shown in Figures 2–7. It should be recognized that the figures are meant to illustrate certain effects and that other model parameters are fixed while the effects of changing a particular parameter are studied.

**Table 1:** Simulation summary

Parameters/methods	Values/descriptions
Gene block standard deviation	
Gene block correlation	
Gene block size	
Noise level	
MV rate	
No. of marker genes	30
No. of total genes	500
Training sample size	60
Testing sample size	200
No. of repetitions for each model	150
Imputation algorithms	RAVG, KNN, LLS.L2, LLS.PC, LS, BPCA
Classification rules	LDA, 3NN, SVM
Feature selection methods	-test, SFFS

#### 3.1.1. Effect of Noise Level

Figure 2 shows the impact of noise level (parameter  in the data model) on imputation and classification. When noise level goes up (from left to right along the -axis), the classification errors (along with the Bayes errors) of the measured dataset and the imputed datasets all increase as expected; the classification errors of the signal dataset stay nearly the same and are consistently the smallest among all the datasets, since the signal dataset is noise-free. Relative to the signal dataset benchmark, the classification performances of imputed datasets deteriorate less than that of the measured dataset as the noise level increases, although their performances degrade with increasing noise. For the smallest noise level, imputation does little to improve upon the measured dataset.

#### 3.1.2. Effect of Variance

The effect of variance (parameter  in the data model) on imputation and classification is shown in Figure 3. As the variance increases, the classification errors of all datasets increase as expected. When the variance is small (e.g., ), all imputed datasets outperform the measured dataset consistently across all the combinations of feature selection methods and classification rules; however, when the variance is relatively large (e.g., ), the measured dataset catches up with and may outperform the datasets imputed by less advanced imputation methods, such as RAVG and KNNimpute. As variance increases, the discriminant power residing in the data is weakened, and the underlying data structure becomes more complex (as confirmed by computing the entropy of the eigenvalues of the covariance matrix of the gene expression matrix [10], data not shown). Thus it becomes harder for the imputation algorithms to estimate the MVs.

In addition, it is observed that the classification performance of one imputed dataset may outperform that of the other imputed dataset for a certain combination of feature-selection method and classification rule, while the performances of the two may reverse for another combination of feature selection and classification rule. For instance, when the classification rule is LDA and the feature selection method is -test, the BPCA imputed dataset outperforms the LLS.L2 imputed dataset; however, the latter outperforms the former when the feature selection method is SFFS and the same classification rule is used (plots on companion website). This suggests that a certain combination of feature-selection method and classification rule may favor one imputation method over another.

#### 3.1.3. Effect of Correlation

Figure 4 illustrates the effect of gene correlation (parameter  in the data model) on imputation and classification. As the gene correlation goes up, the classification errors of all datasets increase as expected. Although it is not straightforward to compare the classification performances of different datasets under different correlations, we notice that the correlation-based MV imputation methods such as LLS.PC and LS may slightly outperform BPCA in larger correlation cases, suggesting that the local correlation structure of a dataset may be better captured by such methods.

#### 3.1.4. Effect of MV Rate

Perhaps the most important observations concern the missing value rate, which is determined by adjusting the parameter  in (6) to obtain a specified percentage  of missing values: . Because we wish to show the effects of two model parameters, we will limit ourselves in the paper to considering 3NN and SVM with -test feature selection. Corresponding results for other cases are on the companion website. Figures 5, 6, and 7 provide the results for the signal standard deviation , and  respectively, with subfigures (a) to (f) of each figure corresponding to noise levels , and , respectively. In all cases, . In Figures 5(a) and 5(b) we observe the following phenomenon: there is improvement on the performance of the various imputation methods as the MV rate initially increases, and then performance deteriorates (quickly, in some cases), as the MV rate continues to increase after a certain point. We shall refer to this phenomenon as the *missing-value rate peaking phenomenon*. It is important to stress that degradation of performance of imputation at larger MV rates is quite noticeable: at 20% the weaker imputation methods perform worse than the measured data and at 25% imputation is detrimental for kNN and not helpful for SVM. In Figures 5(c) and 5(d) we again observe the MV rate peaking phenomenon; however, imputation performs better relative to the measured data. Imputation remains better throughout for SVM and only gets worse for kNN at MV rate 25%. In Figures 5(e) and 5(f) the peaking phenomenon is again noticeable, but for this noise level imputation is much better relative to the measured data and all imputation methods remain better at all MV rates. Similar trends are observed in Figures 6 and 7, the difference being that as  increases from  to  and , the imputation methods perform increasingly worse with respect to the measured data. Note particularly the degraded performance of the simpler imputation schemes.

**Figure 8:**
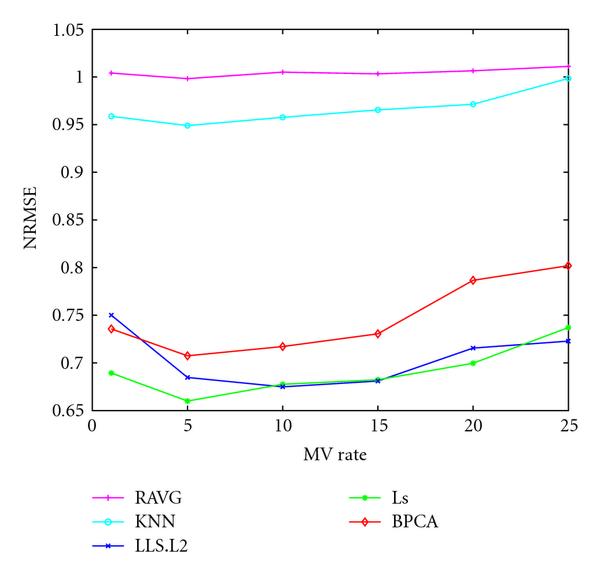
**The NRMSE values (-axis) of the five imputation algorithms with respect to the MV rate (-axis)**. The underlying distribution parameters are: SD , noise level , gene correlation .

Figure 8 displays the behavior of NRMSE as a function of MV rate. Here, we also observe a peaking phenomenon for the NRMSE, though a modest one. This is in contrast to previous studies, which all generally report the NRMSE to increase monotonically with increasing MV rate [4, 5, 9, 13]; this may be a consequence of the different way in which the MVs are selected in those studies as compared with the present one; in the former, MVs are picked randomly, whereas in the latter, MVs are picked based on quality considerations, revealing the peaking phenomenon.

### 3.2. Results for the Patient Data

**Figure 9:**
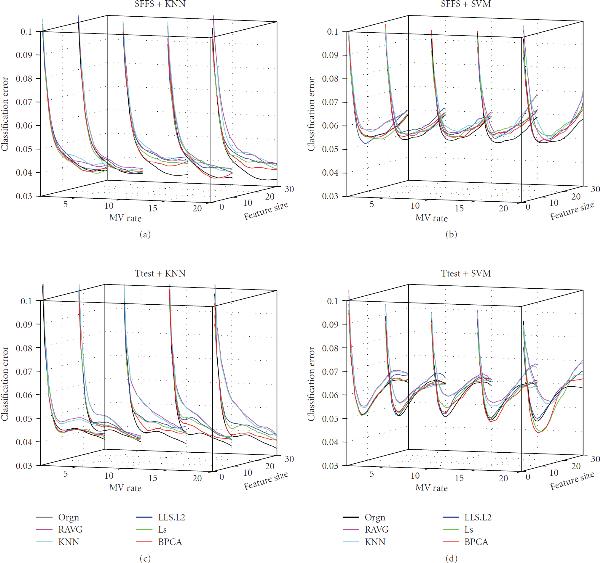
**The classification errors of the measured prostate cancer dataset (orgn), and the five imputed datasets**. Each panel in the figure corresponds to one combination of the feature selection methods and the classification rules, which is given by the title. The -axis labels the number of selected genes, the -axis is the MV rate, and the -axis is the classification error.

**Figure 10:**
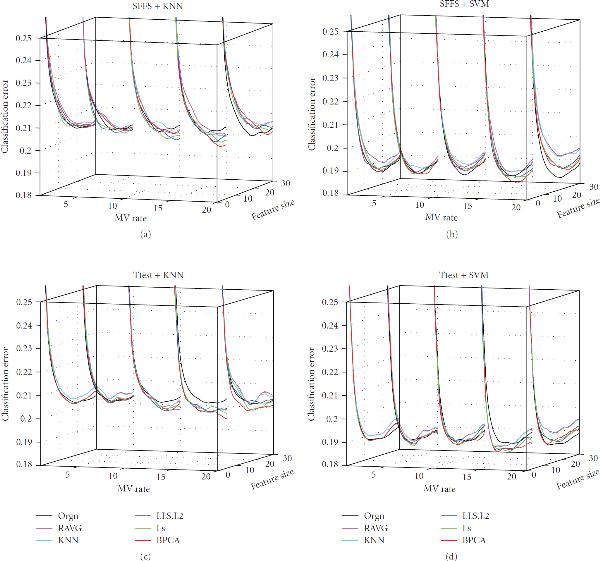
**The classification errors of the measured breast cancer dataset (orgn) and the five imputed datasets**. The meanings of the axes and titles are the same as in Figure 9.

For the patient data, since the true signal is unknown, we only conduct the comparison of imputations with respect to different MV rates. The effect of MV rate is shown in Figures 9 and 10, for the BREAST and the PROST dataset, respectively. The trends observed are similar to those in the synthetic data study, in the sense that there is a degradation of performance of imputation methods with increasing MV rates. On the other hand, the missing-value rate peaking phenomenon is less evident here, but still present, as can be seen from the fact that the classification performance of LLS, LS, and BPCA imputed datasets in a few cases becomes better under a larger MV rate than the corresponding datasets with a smaller MV rate.

It is again observed that the classification performances of imputed datasets depend on the underlying combination of feature selection method and classification rule. For example, RAVG and KNNimpute show satisfactory performances for the combinations SFFS + LDA and Ttest + LDA (data not shown) but perform relatively poorly for the other combinations.

**Figure 11:**
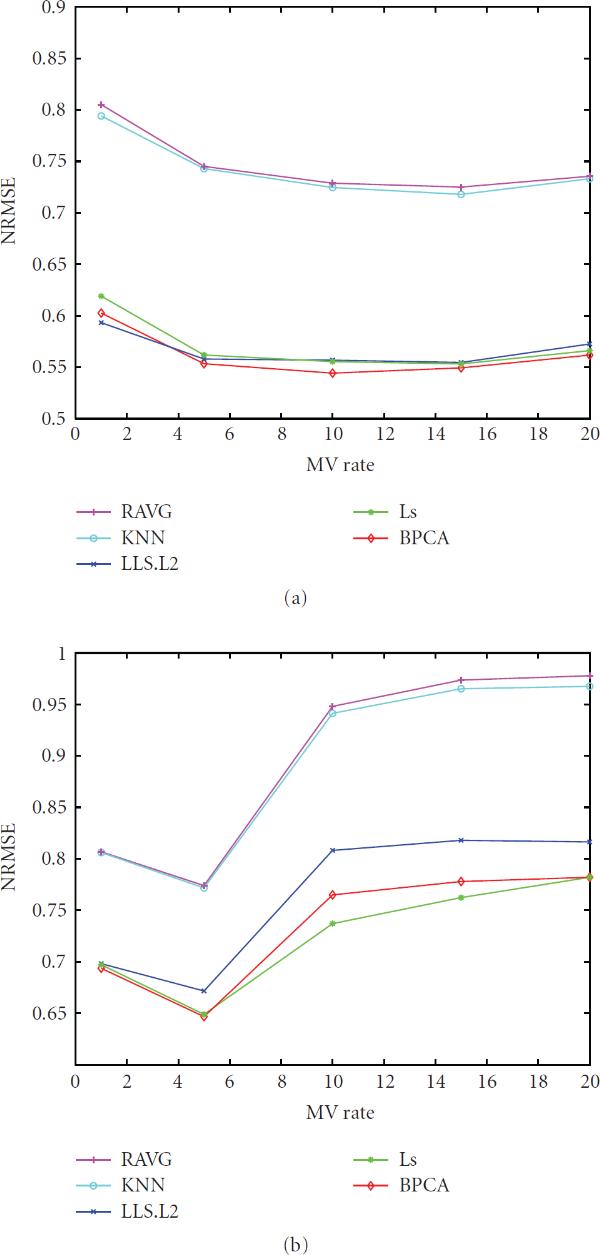
The NRMSE values (-axis) of the five imputation algorithms with respect to the MV rate (-axis) for the PROST dataset and the BREAST dataset

The NRMSE values of different imputation methods generally decrease first and then increase as the MV rate increases (see Figure 11) which is similar to the trend observed in synthetic data study.

It is also found that there is no strong correlation between the low-level performance measure NRMSE and the high-level measure classification error. A small NRMSE may not necessarily suggest a small classification error, that is, an imputation method may perform better than another imputation method in terms of estimation accuracy, but the former may not be as good as the latter in terms of classification performance. In other words, although a given imputation method may be more accurate than another when measured by NRMSE, it might decrease more the discrimination power presents in the original data.

## 4. Conclusions

We study the effects of MVs and their imputation on classification by using a model-based approach. The model-based approach is employed because it enables systematic study of the complicated microarray data analysis pipeline, including imputation, feature selection and classification. Moreover, it gives us ground truth for the differentially expressed genes, allowing the computation of imputation accuracy and classification error. We also carry out a simulation using real patient data from two cancer studies to complement the findings of the synthetic data study.

Our results suggest that it is beneficial to apply MV imputation on the microarray data when the noise level is high, variance is small, or gene-cluster correlation is strong, under small to moderate MV rates. In these cases, if data quality metrics are available, then it may be helpful to consider the data point with poor quality as missing and apply one of the most robust imputation algorithms, such as LLS, and BPCA, to estimate the true signal based on the available high-quality data points, in which case the classifier designed on the imputed dataset with reduced noise may yield better error rates than the one designed on the original dataset.

However, at large MV rates, we observed that imputation methods are NOT recommended, and the original measured data yields better classification performance. Regarding MV rate, our results indicate the presence of a peaking phenomenon: performance of imputation methods actually improves initially as the MV rate increases, but after an optimum point is reached, performance quickly deteriorates with increasing MV rates. This was observed very clearly in the synthetic data simulation, and less so with the patient data, even though the phenomenon is still noticeable.

As for the NRMSE criterion, which is the figure of merit employed by most studies, we also observe a peaking phenomenon with increasing MV rate, in contrast to previous studies that report the NRMSE to increase monotonically with increasing MV rate; this may be a consequence of the different ways in which the MVs are selected in those studies as compared with the present one; in the former, MVs are picked randomly, whereas we pick MVs based on quality considerations.

## References

[B1] de Brevern AG Hazout S Malpertuy A Influence of microarray experiments missing values on the stability of gene groups by hierarchical clustering BMC Bioinformatics 2004 5 article 114 10.1186/1471-2105-5-114 PMC514701 15324460

[B2] Troyanskaya O Cantor M Sherlock G Missing value estimation methods for DNA microarrays Bioinformatics 2001 17 6 520 525 10.1093/bioinformatics/17.6.520 11395428

[B3] Oba S Sato M-A Takemasa I Monden M Matsubara K-I Ishii S A Bayesian missing value estimation method for gene expression profile data Bioinformatics 2003 19 16 2088 2096 10.1093/bioinformatics/btg287 14594714

[B4] Bø TH Dysvik B Jonassen I LSimpute: accurate estimation of missing values in microarray data with least squares methods Nucleic Acids Research 2004 32 3 e34 10.1093/nar/gnh026 14978222 PMC374359

[B5] Kim H Golub G Park H Missing value estimation for DNA microarray gene expression data: local least squares imputation Bioinformatics 2005 21 2 187 198 10.1093/bioinformatics/bth499 15333461

[B6] Jörnsten R Wang H Welsh W Ouyang M DNA microarray data imputation and significance analysis of differential expression Bioinformatics 2005 21 22 4155 4161 10.1093/bioinformatics/bti638 16118262

[B7] Scholz M Kaplan F Guy CL Kopka J Selbig J Non-linear PCA: a missing data approach Bioinformatics 2005 21 20 3887 3895 10.1093/bioinformatics/bti634 16109748

[B8] Tuikkala J Elo L Nevalainen O Aittokallio T Improving missing value estimation in microarray data with gene ontology Bioinformatics 2006 22 5 566 572 10.1093/bioinformatics/btk019 16377613

[B9] Hu J Li H Waterman MS Zhou XJ Integrative missing value estimation for microarray data BMC Bioinformatics 2006 7 article 449 10.1186/1471-2105-7-449 PMC1622759 17038176

[B10] Brock GN Shaffer JR Blakesley RE Lotz MJ Tseng GC Which missing value imputation method to use in expression profiles: a comparative study and two selection schemes BMC Bioinformatics 2008 9 article 12 10.1186/1471-2105-9-12 PMC2253514 18186917

[B11] Sehgal M Gondal I Dooley LS Coppel R How to improve postgenomic knowledge discovery using imputation EURASIP Journal on Bioinformatics and Systems Biology 2009 2009 14 10.1155/2009/717136 PMC3171441 19223972

[B12] Scheel I Aldrin M Glad IK Sorum R Lyng H Frigessi A The influence of missing value imputation on detection of differentially expressed genes from microarray data Bioinformatics 2005 21 23 4272 4279 10.1093/bioinformatics/bti708 16216830

[B13] Tuikkala J Elo LL Nevalainen OS Aittokallio T Missing value imputation improves clustering and interpretation of gene expression microarray data BMC Bioinformatics 2008 9 article 202 10.1186/1471-2105-9-202 PMC2386492 18423022

[B14] Wang D Lv Y Guo Z Effects of replacing the unreliable cDNA microarray measurements on the disease classification based on gene expression profiles and functional modules Bioinformatics 2006 22 23 2883 2889 10.1093/bioinformatics/btl339 16809389

[B15] Shi Y Cai Z Lin G Mandoiu I, Zelikovsky A Classification accuracy based microarray missing value imputation Bioinformatics Algorithms: Techniques and Applications 2007 Wiley-Interscience, Hoboken, NJ, USA 303 328

[B16] Hoyle DC Rattray M Jupp R Brass A Making sense of microarray data distributions Bioinformatics 2002 18 4 576 584 10.1093/bioinformatics/18.4.576 12016055

[B17] Autio R Kilpinen S Saarela M Kallioniemi O Hautaniemi S Astola J Comparison of Affymetrix data normalization methods using 6,926 experiments across five array generations BMC Bioinformatics 2009 10 supplement 1 article S24 10.1186/1471-2105-10-S1-S24 PMC2648747 19208124

[B18] Kerr M Martin M Churchill GA Analysis of variance for gene expression microarray data Computational Biology 2001 7 6 819 837 10.1089/10665270050514954 11382364

[B19] Kerr M Martin M Churchill GA Statistical design and the analysis of gene expression microarray data Genetical Research 2001 77 2 123 128 11355567 10.1017/s0016672301005055

[B20] Attoor S Dougherty ER Chen Y Bittner ML Trent JM Which is better for cDNA-microarray-based classification: ratios or direct intensities Bioinformatics 2004 20 16 2513 2520 10.1093/bioinformatics/bth272 15454406

[B21] Tseng GC Oh M-K Rohlin L Liao JC Wong WH Issues in cDNA microarray analysis: quality filtering, channel normalization, models of variations and assessment of gene effects Nucleic Acids Research 2001 29 12 2549 2557 10.1093/nar/29.12.2549 11410663 PMC55725

[B22] Shmulevich I Zhang W Binary analysis and optimization-based normalization of gene expression data Bioinformatics 2002 18 4 555 565 10.1093/bioinformatics/18.4.555 12016053

[B23] Yang YH Dudoit S Luu P Normalization for cDNA microarray data: a robust composite method addressing single and multiple slide systematic variation Nucleic Acids Research 2002 30 4 article e15 10.1093/nar/30.4.e15 PMC100354 11842121

[B24] Quackenbush J Microarray data normalization and transformation Nature Genetics 2002 32 5 496 501 12454644 10.1038/ng1032

[B25] Hua J Waibhav T Dougherty ER Performance of feature-selection methods in the classification of high-dimension data Pattern Recognition 2009 42 3 409 424 10.1016/j.patcog.2008.08.001

[B26] Brás LP Menezes JC Dealing with gene expression missing data IEE Proceedings Systems Biology 2006 153 3 105 119 10.1049/ip-syb:20050056 16984085

[B27] Nguyen D Wang N Carroll R Evaluation of missing value estimation for microarray data Journal of Data Science 2004 2 347 370

[B28] Duda R Hart P Pattern Classification 2001 John Wiley & Sons, New York, NY, USA

[B29] Pudil P Novovicova J Kittler J Floating search methods in feature selection Pattern Recognition Letters 1994 15 11 1119 1125 10.1016/0167-8655(94)90127-9

[B30] Sima C Dougherty ER The peaking phenomenon in the presence of feature-selection Pattern Recognition Letters 2008 29 11 1667 1674 10.1016/j.patrec.2008.04.010

[B31] Sima C Attoor S Brag-Neto U Lowey J Suh E Dougherty ER Impact of error estimation on feature selection Pattern Recognition 2005 38 12 2472 2482 10.1016/j.patcog.2005.03.026

[B32] Kudo M Sklansky J Amin A, Dori H, Pudil P Classifier-independent feature selection for two-stage feature selection Proceedings of the Joint IAPR International Workshops on Advances in Pattern Recognition, Lecture Notes in Computer Science 1998 1451 Springer, Berlin, Germany 548 554

[B33] Veer L Dai H Vijver M Gene expression profiling predicts clinical outcome of breast cancer Nature 2002 415 6871 530 536 10.1038/415530a 11823860

[B34] Vijver M He Y Veer L A gene-expression signature as a predictor of survival in breast cancer The New England Journal of Medicine 2002 347 25 1999 2009 10.1056/NEJMoa021967 12490681

[B35] Lapointe J Li C Higgins JP Gene expression profiling identifies clinically relevant subtypes of prostate cancer Proceedings of the National Academy of Sciences of the United States of America 2004 101 3 811 816 10.1073/pnas.0304146101 14711987 PMC321763

[B36] Weng L Dai H Zhan Y He Y Stepaniants SB Bassett DE Rosetta error model for gene expression analysis Bioinformatics 2006 22 9 1111 1121 10.1093/bioinformatics/btl045 16522673

